# Identification of Circular RNA Expression Profiles in White Adipocytes and Their Roles in Adipogenesis

**DOI:** 10.3389/fphys.2021.728208

**Published:** 2021-08-19

**Authors:** Peng-peng Zhang, Qiu Han, Ming-xuan Sheng, Chun-yu Du, Ya-ling Wang, Xiao-fang Cheng, Hai-xia Xu, Cen-cen Li, Yong-jie Xu

**Affiliations:** ^1^Department of Biotechnology, College of Life Sciences, Xinyang Normal University, Xinyang, China; ^2^Institute for Conservation and Utilization of Agro-Bioresources in Dabie Mountains, Xinyang Normal University, Xinyang, China

**Keywords:** circRNA, adipocyte, adipogenesis, obesity, high-throughout RNA sequencing

## Abstract

Obesity and its related metabolic diseases have become great public health threats worldwide. Although accumulated evidence suggests that circRNA is a new type of non-coding RNAs regulating various physiological and pathological processes, little attention has been paid to the expression profiles and functions of circRNAs in white adipose tissue. In this study, 3,771 circRNAs were detected in three stages of white adipogenesis (preadipocyte, differentiating preadipocyte, and mature adipocyte) by RNA-seq. Experimental validation suggested that the RNA-seq results are highly reliable. We found that nearly 10% of genes which expressed linear RNAs in adipocytes could also generate circRNAs. In addition, 40% of them produced multiple circRNA isoforms. We performed correlation analysis and found that a great deal of circRNAs (nearly 50%) and their parental genes were highly correlated in expression levels. A total of 41 differential expression circRNAs (DECs) were detected during adipogenesis and an extremely high ratio of them (80%) were correlated with their parental genes, indicating these circRNAs may potentially play roles in regulating the expression of their parental genes. KEGG enrichment and GO annotation of the parental genes suggesting that the DECs may participate in several adipogenesis-related pathways. Following rigorous selection, we found that many up-regulated circRNAs contain multiple miRNAs binding sites, such as miR17, miR-30c, and miR-130, indicating they may potentially facilitate their regulatory functions by acting as miRNA sponges. These results suggest that plenty of circRNAs are expressed in white adipogenesis and the DECs may serve as new candidates for future adipogenesis regulation.

## Introduction

Obesity is recognized as one of the severe threats to public health due to its strong positive association with various diseases, including diabetes, hypertension, cardiovascular diseases, and even cancers (Blüher, 2019). Obesity is characterized by the accumulation of white adipose tissue, which is dependent on an increase of adipocyte number (adipogenesis) and enlargement of adipocytes (hypertrophy) (Stefan, 2020). One possible approach to prevent obesity is to reduce adipocyte number, but a better understanding of the regulators controlling adipogenesis is needed. In the last few decades, scientists have identified many key protein-encoding genes, such as PPARγ and C/EBP family genes, which are essential for adipogenesis (Rosen et al., 2000). Despite that, there has been a rapidly growing interest in the role of non-coding RNAs in adipogenesis. A larger number of microRNAs and long-non-coding RNAs have been reported to play vital roles in adipogenesis (Sun et al., 2013; Arner and Kulyté, 2015; Lorente-Cebrián et al., 2019). In recent years, circRNA is emerging as another type of non-coding RNA, with important functions in physiological systems and disease contexts.

CircRNA is a type of covalently closed and single-stranded RNA, which is produced by back-splicing of pre-mRNA (Li et al., 2018). It was first reported in the 1990s. However, it is considered as an abnormal splicing product with little function. Until recently, with the progress of high throughout technology, it has been revealed that circRNAs are widely expressed across all eukaryotic species and participate in regulating various biological activities (Kristensen et al., 2019; Patop et al., 2019). CircRNAs can act as miRNA sponges or protein decoys to regulate transcription, splicing, and RNA stability (Hansen et al., 2013; Conn et al., 2017; Du et al., 2017). Some circRNAs may even encode functional proteins by IRES-driven mechanisms (Fan et al., 2019). Through the above ways, circRNAs control many cellular processes such as cell proliferation, differentiation, and apoptosis, which are deeply related to correct tissue development and proper tissue functions. CircRNAs have been extensively studied in the organogenesis of various human organs, such as the central nervous system, cardiovascular system, and skeletal muscle (Khan et al., 2016; Piwecka et al., 2017; Zhang P. et al., 2019). It is also reported that abnormal expression of circRNAs leads to human disease, including cancers and Alzheimer’s disease (Haque and Harries, 2017).

Unlike the central nervous system and cancers, fewer studies have aimed to define circRNA function in adipose tissue. Arcinas et al. (2019) performed global circRNA profiles in both epididymal and inguinal fat of humans and mice, they identified thousands of adipose circRNAs. Liu et al. (2020) tried to identify differentially expressed adipose circRNAs from obese and lean individuals. Otherwise, Zhang H. et al. (2019) reported that exosomal circRNAs, which were derived from a gastric tumor, could regulate white adipose browning. Liu et al. (2018) analyzed the expression patterns of circRNAs during porcine subcutaneous preadipocyte differentiation. However, the expression of circRNAs in the process of mouse white adipogenesis remains unknown.

The majority of research investigates adipogenesis molecular pathways was performed *in vitro* using cell lines, e.g., 3T3-L1 or C3H/10T1/2 (Bahmad et al., 2020). However, their ability to differentiate *in vivo* is limited. An alternative approach is the use of primary preadipocytes. Cells isolated from WAT stromal vascular fraction (SVF) can differentiate into mature adipocytes. Regardless some cells are included in SVF other than preadipocytes, such as endothelial cells, pericytes, and fibroblasts, it may more accurately represent adipose tissue function *in vivo* (Rodeheffer et al., 2008), thus WAT SVF is a widely used model to study adipogenesis *in vitro*.

The main goal of the current study was to determine the circRNA profiles during adipogenesis. We isolated SVF cells from mouse white adipose tissue and identify circRNAs by RNA-seq. We discovered a lot of novel circRNAs and characterized their expression profiles in the process of adipogenesis. Furthermore, we identified differential expression circRNAs (DECs) and determined their correlation with the corresponding parental genes. The miRNA binding sites of circRNAs were predicted, suggesting the potential roles of circRNAs in adipogenesis.

## Materials and Methods

### Animals

Mice were bought from the Model Animal Research Centre of Nanjing University in a C57BL/6J background. All the experiments involving mice were guided by the Xinyang Normal University Animal Care and Use Committee.

### Cell Culture

Primary white adipose SVF cells were cultured as we described previously (Shan et al., 2016). Briefly, the inguinal fat pad was collected from 6-week-old female mice and washed with PBS twice. Then, the fat pad was minced with scissors and digested with collagenase type I (1.5 mg/ml, #SCR103, Sigma-Aldrich) at 37°C for 40 min. When the digestion was finished, the growth medium contained 85% high glucose DMEM medium (#11965126, Thermo Fisher Scientific) and 15% fetal bovine serum (#10099141, Thermo Fisher Scientific) was added to dilute the collagenase. The tissue debris was removed through a 70-μm cell strainer. The medium was subjected to centrifuge to get SVF cells pellet. SVF cells were resuspended with the growth medium. When the cells reached 90% confluence, they were induced to adipogenesis, with a cocktail containing DMEM, 10% fetal bovine serum, 2.85 mM recombinant human insulin (#I8830, Solarbio), 0.3 mM dexamethasone (#D8040, Solarbio), and 0.63 mM 3-isobutyl-methylxanthine (#I7018, Sigma-Aldrich). After 4 days, the cocktail was switched to a DMEM medium supplemented with 10% fetal bovine serum, 10 nM triiodothyronine (T3, #T6397, Sigma-Aldrich), and 200 nM insulin to induce mature adipocytes.

### Total RNA Preparation and RNA-Sequencing

Total RNA was purified from adipocytes using Trizol Reagent (#15596026, Thermo Fisher Scientific). To enrich circRNAs, the rRNA was removed with Ribo-zero rRNA Removal Kit (#RZH1046, Epicentre) and linear RNA was digested with RNase R (#RNR07250, Epicentre). Then, the sequencing libraries were prepared by RNA Library Prep Kit (#E7760S, NEB) and sequenced on an illumine platform. Raw datasets have been deposited at the Gene Expression Omnibus (#GSE178502).

### Identification of CircRNA

The circRNA was identified as previously described (Zhang P. et al., 2018). First, the adapter reads and low-quality reads were removed using Fastp (version 0.20.1) (Chen S. et al., 2018). Then, the clean data were mapped to the reference mouse genome mm9 using BWA-MEM (version 0.7.17) (Li, 2013). Subsequently, circRNAs were identified using CIRI2 (Gao et al., 2018). The expression levels of circRNAs were measured by “circRNA counts per million circRNA reads” (circCPM) (Shao et al., 2019). Then, DECs were detected by DESeq2 (version 1.10.1) with a likelihood ratio test (Love et al., 2014). Expression patterns of the DECs were obtained by using degPatterns function from the R package DEGreport (version 1.28.0) (Pantano, 2021). The degPatterns function was run using the default parameters, except that the minimum number of circRNAs in each group was set to 1 (minc = 1).

### qPCR Analysis

Random primers and Reverse Transcription Kit (#RR037A, Takara) were used to obtain cDNA according to the manufacturer’s protocol. CircPrimer 2.0 software was used to annotate and obtain circRNA sequences (Zhong et al., 2018). Then, the divergent primers, which coved the back-splicing regions, were designed by Primer3^1^ (Untergasser et al., 2012). The PCR products of divergent primers were sequenced to validate the corresponding back-splicing sites. The relative expression levels of selected circRNAs were detected by qRT-PCR using TB Green Premix Ex II (#RR820A, Takara) on a LightCycler 96 system (Roche, Germany) according to the instructions. 18S was used to normalize the threshold cycle (Ct) values, and gene expression was quantified using the relative quantitation method (2^–ΔΔCt^). All experimental data are presented as means ± SD.

### GO and KEGG Pathway Analyses

The parental genes of circRNAs were subjected to functional annotation. ClusterProfiler package in Bioconductor was used to perform GO analysis (Yu et al., 2012) and *q*-values < 0.05 were considered statistically significant. KEGG pathways were enriched by KOBAS online software^2^ (Bu et al., 2021) and the corrected *P*-values < 0.05 were considered statistically significant.

### Correlation Analyses Between CircRNAs and Their Parental Genes

To examine the correlation between each circRNA and the parental gene, expression levels of mRNA were extracted from our previous study (GEO accession number GSE173710). Then the average expression levels of circRNA and mRNA on D0, D4, and D8 were used to calculate the correlation by using the Pearson correlation test and the *P*-values < 0.05 were considered statistically significant.

### Construction of the CircRNA-miRNA Network

The circRNA-miRNA interactions were predicted using miRDB with a predicted score over 85 (Chen and Wang, 2020). Then, the circRNA-miRNA network was constructed using Cytoscape 3.8.2 (Shannon et al., 2003).

## Results

### Identification of CircRNAs in Growth and Differentiation WAT Adipocytes

**FIGURE 1:**
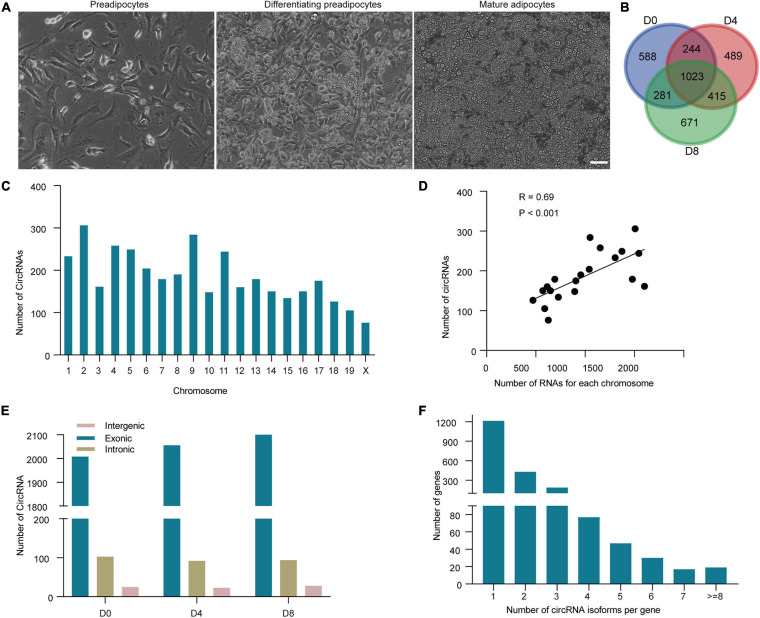
Identification and characterization of circRNAs in white adipogenesis. **(A)** Representative pictures of WAT adipocytes during adipogenesis. Scale bar, 20 μm. **(B)** Identification of circRNAs at each adipogenesis stage. **(C)** Distribution of circRNAs on each chromosome. **(D)** Association of circRNAs and mRNA numbers from the same chromosome. **(E)** Genomic origins of the circRNA type in each adipogenesis stage. **(F)** The number of circRNAs derived from per gene.

To identify circRNAs in adipogenesis, RNA was collected from WAT SVF on day 0 (D0), day 4 (D4), and day 8 (D8) post differentiation, corresponding to the proliferation, premature and mature stages of WAT adipocytes differentiation, with two biological replicates for each stage (Figure 1A). To enrich circRNA, the rRNA and linear RNA were removed. Then the RNA samples were subjected to RNA-seq. The CIRI2 was used to predict *de novo* circRNAs. As circRNAs identified between replicates are usually showed low consistency, we kept the circRNAs with a minimum of two reads identified in both two replicates. A total of 3,711 circRNAs were identified (Supplementary Table 1). Compared to the publicly available circBase database,^3^ we found 1,324 circRNAs were novel (35.11%). As shown in Figure 1B, circRNAs were identified on D0, D4, and D8, respectively. It is noticed that 1,023 circRNAs (27.13%) were continually expressed in all stages of adipogenesis, while 588, 489, and 671 circRNAs were only detected on D0, D4, and D8 respectively, indicating the stage-specific expression of circRNAs.

### Characteristics of the Adipocyte CircRNAs

We analyzed the chromosome distribution of the circRNAs. We noticed that chromosome 2 generated the greatest number of circRNAs, while chromosome X was the least (Figure 1C). Considering that chromosome X is the shortest, we calculated the relationship between circRNA number and chromosome length. The results showed that the correlation was significant (*R* = 0.69, *P* < 0.001). As circRNAs share pre-RNAs with mRNAs, we further analyzed the correlation between linear mRNA number and circRNA number in each chromosome, and a much higher correlation was found (*R* = 0.77, *P* < 0.05; Figure 1D), indicating that the generation of circRNAs may associate with linear mRNAs.

Upon the genomic origin of junction sites, circRNAs can be classified into exonic, intronic, and intergenic circRNA. As described in Figure 1E, the ratio of circRNA types was similar in all the stages. The majority of the circRNAs were derived from protein-coding exons (94.53%) of circRNAs. The other circRNAs were derived from introns or intergenic regions. Our previous study showed that 20,703 mRNAs could be detected during white adipogenesis (with a minimum of two reads in both two replicates, accession number GSE173710). We found nearly 10% (2,018 genes) of them can generate circRNAs. Further, we found that a great deal of these parental genes (about 40%) gave rise to more than one type of circRNA isoforms. Arhgap10 even produced up to 20 distinct circRNA isoforms (Figure 1F). The above results suggest that alternative splicing is very common in circRNA biogenesis, thus expand the diversity of circRNA expression profiles in adipogenesis.

### Experimental Validation of the Predicted CircRNAs

**FIGURE 2:**
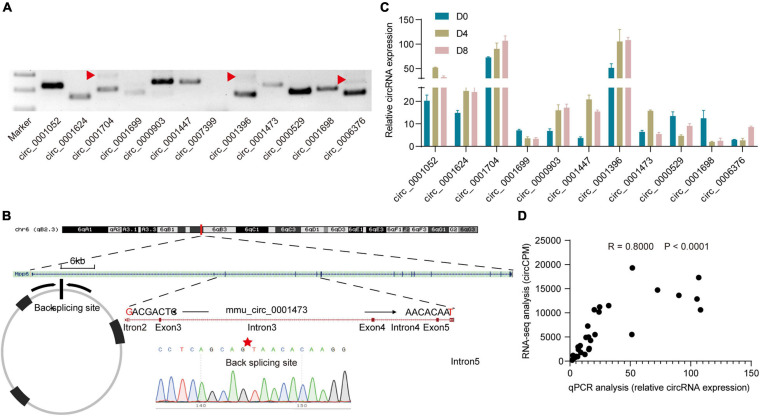
Verification of circular RNAs. **(A)** Electrophoretic band of circRNAs. The red triangle represents double-size products. **(B)** Representative Sanger sequencing results of circRNAs. The red star represents the back-splicing site. **(C)** Relative circRNA expression levels detected by qPCR (*n* = 3). **(D)** Correlation analysis of qPCR results and RNA-seq results.

To confirm the authenticity of the RNA-seq results, we randomly chose 12 circRNAs and designed divergent primers (primers are list in Supplementary Table 2). As shown in Figure 2A, 11 of the 12 circRNAs were successfully amplified. In some of the cases, double products were detected which may be generated by multiple rounds of RT around a circular RNA template (Danan et al., 2012). Further, Sanger sequence results detected the expected back-splicing sites (Figure 2B). Next, we checked circRNAs expression levels of the 11 circRNAs by qPCR (Figure 2C). Then the correlation between the RNA-seq results and qPCR results was examined. We found a strong correlation between them (*R* = 0.800, *P* < 0.0001; Figure 2D). The above results suggested that RNA-seq results are reliable.

### Differential Expression of CircRNA During Adipogenesis

**FIGURE 3:**
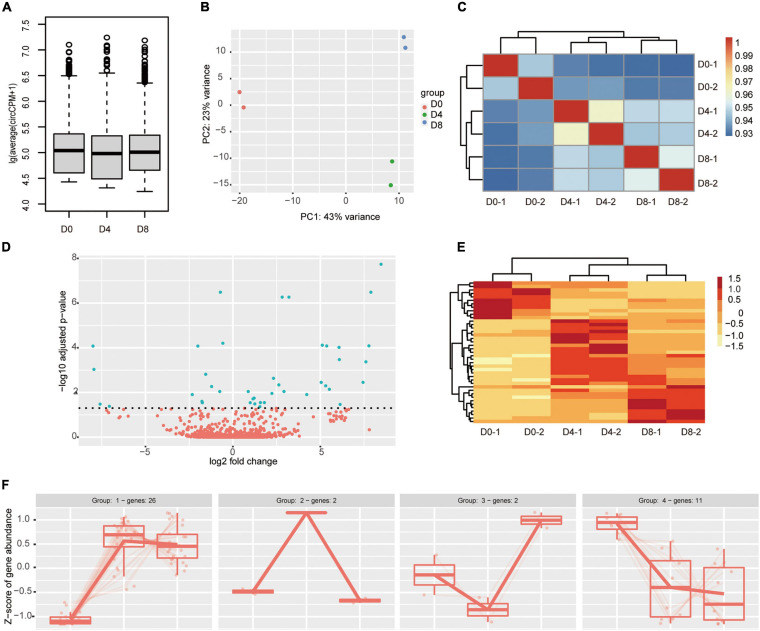
Differential circRNAs expression during adipogenesis. **(A)** Relative expression abundance of circRNAs during adipogenesis (circCPM). **(B)** Principal component analysis (PCA) plot of RNA samples. **(C)** Hierarchical clustering analyses of samples correlation using DESeq2 rlog-normalized RNA-seq results. **(D)** Volcano plot comparing circRNAs abundance between different adipogenesis stages. The green color indicates the differentially expressed circRNAs (padj < 0.05), while the red color indicates not significant change circRNAs. **(E)** Heatmap showing differentially expressed circRNAs across different stages of adipogenesis. **(F)** Expression patterns of the differential expressed circRNAs during adipogenesis.

To compare expression levels of circRNA between different stages, we first checked the overall expression of circRNAs with the boxplot. As shown in Figure 3A, the average abundance of circRNAs in all the three stages of adipogenesis was comparable to each other. To explore the similarity of the samples, we performed principal component analysis. As indicated in Figure 3B, the distance between two biological replicates was very close to each other, indicating high repeatability. Meanwhile, the D0 group was located far away from the other groups, suggesting a great difference in circRNA expression patterns between the proliferation and differentiation stages. Consistent with the principal component analysis, the hierarchical tree also showed biological replicates were highly correlated with each other (Figure 3C). Subsequently, we identified DECs across adipogenesis by DESeq2 with the Likelihood ratio test. We set the cut-off as padj < 0.05. Only 41 DECs were identified. The majority of them were upregulated (28 of 41) and 13 were downregulated (Figure 3D and Supplementary Table 3). Consistent with the above results, the heatmap showed marked differences between the proliferation stage and the differentiation stages (Figure 3E). As many circRNAs regulate the expression of their parental genes, the roles of circRNAs may be revealed through functional analysis of their parental genes. Despite the two circRNAs fell outside the genomic regions of annotated genes, the parental genes of the other 39 DECs were used. KOBAS gene-list enrichment showed that many adipogenesis and fat metabolism pathways were significantly enriched, such as GnRH signaling pathway, MAPK signaling pathway, type II diabetes mellitus, calcium signaling pathway, and cAMP signaling pathway. GO annotations indicated that calcium channels, actinin binding, and transmembrane receptor protein kinase activity were significantly enriched (Supplementary Table 4).

We further examined the expression patterns of the 41 DECs using DegPatterns function of R package DEGreport. A total of four groups were identified (Figure 3F and Supplementary Table 3). The circRNA numbers ranged from 2 to 26 in the four groups. Group 1 was the largest, which contained 26 circRNAs. In group 1, the circRNAs showed increased expression levels in the differentiation stages compared to the proliferation stage. In contrast to group 1, group 4 showed an opposite trend, the circRNAs decreased in the differentiation stages. Both group 2 and group 3 contained only two circRNAs. Group 2 showed a transient increase on D4 followed by a decrease. Group 3 showed a transient decrease on D4 followed by a sharp increase. To annotate the role of the circRNAs in group 1 and group 4. We performed GO and KEGG analysis (Supplementary Table 5). Unfortunately, few GO terms were significantly enriched. The results showed four GO terms were enriched in group 1, they were transmembrane receptor protein kinase activity, transmembrane receptor protein serine/threonine kinase activity, activin binding, growth factor binding, and growth factor binding. In group 4, actinin binding and alpha-actinin binding were enriched. KOBAS enrichment showed no pathway was significantly enriched in group 4 and only a few pathways were enriched in group 1, such as propanoate metabolism, MAPK signaling pathway, GnRH signaling pathway, and TGF-beta signaling pathway.

### Correlation of the Expression Between CircRNAs and Linear RNAs

To evaluate the change of circRNAs expression and their parental genes between different stages of adipogenesis. The expression data of mRNA counterparts were collected from our previous study (GEO accession number GSE173710). We tried to calculate the overall correlation between circRNAs expression and their parental genes, but no significant correlation was found. However, when we checked the expression of individual circRNA and the parental gene in adipogenesis, we identified 1,806 circRNA-mRNA pairs (48.67%) that were significantly correlated with each other, including 1,379 (37.16%) positively correlation and 427 (11.51%) negatively correlation (Supplementary Table 6).

**FIGURE 4:**
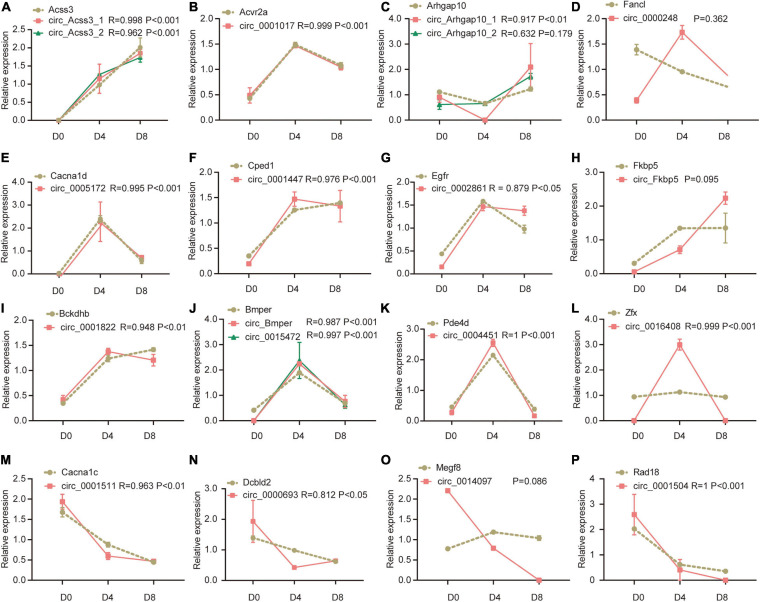
Representative results of correlation analysis between differentially expression circRNAs and their parental genes during adipogenesis. For the convenience of drawing both circRNA and mRNA expression in the figure, the relative expression level was used (expression level at each time point was divided by its average expression level across adipogenesis). **(A–P)** The correlation between Acss3, Acvr2a, Arhgap10, Fancl, Cacna1d, Cped1, Egfr, Fkbp5, Bckdhb, Bmper, Pde4d, Zfx, Cacna1c, Dcbld2, Megf8, Rad18, and their corresponding circRNAs.

We further analyzed the correlation between the 39 DECs and their linear counterparts. We found 33 of the DECs were correlated with their linear counterparts. Interestingly, all of them showed positive correlation, ranging from 0.812 to 0.999 (*P* < 0.05, Figure 4 showed the representative results and the other results could be found in Supplementary Figure 1). Notably, circRNA generated by Acvr2a showed almost the same trend as the linear counterpart (*R* = 0.999), while circRNAs generated by Fancl and Megf8 were not significantly correlated with their linear counterparts. In Figure 4D, the expression of Fancl linear counterpart continuously decreased in the process of adipogenesis, while the expression of circRNA showed a transient increase on D4 followed by a decrease on D8. As shown in Figure 4A, both two circRNA isoforms generated by Acss3 were highly correlated with the linear counterpart. But in Figure 4C, only one of the circRNA isoforms generated by Arhgap10 was significantly correlated with the linear counterpart. In Figure 4L, it seemed that the expression of the Zfx linear counterpart and the circRNA were not correlated. However, after we inspected the data, we found that the Zfx linear counterpart increased by 20% on D4, then it decreased to a similar level as D0. The corresponding circRNA showed the same expression pattern despite much more change on D4, hence they showed a high correlation. In summary, these results indicated that many of the circRNAs are highly correlated with their linear counterparts. CircRNAs may be potentially involved in the regulation of linear RNA expression in adipogenesis.

### Potential CircRNA-miRNA Interaction Network

**FIGURE 5:**
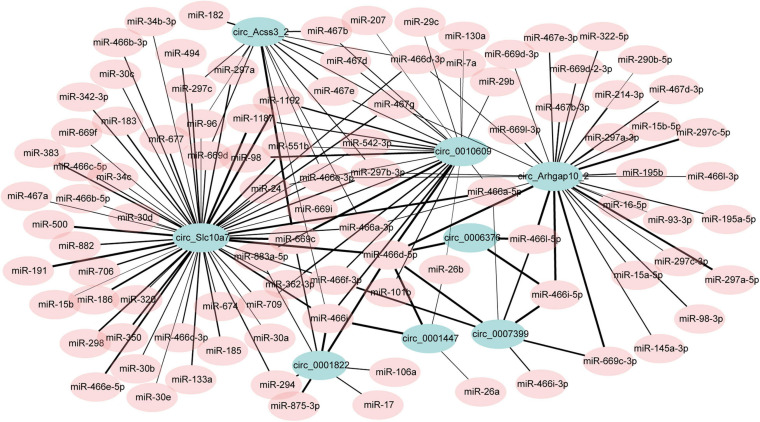
The potential circRNA-miRNA interaction network. The width of the edge indicates the prediction score (range from 85 to 100).

CircRNAs may affect gene expression by interacting with miRNAs (Zhong Q. et al., 2019). In the up-regulated circRNAs, we chose the top 15 highly expressed circRNAs. The potential miRNA binding sites of these circRNAs were predicted using miRDB. As some of the circRNAs may not express in adipocytes, we filtered them according to the previous data studying miRNA profiles in adipogenesis (GEO accession: GSE75697). Then, 8 of the15 circRNAs were left, which contain many miRNA binding sites. A total of 148 circRNA-miRNA interactions were identified with a predicted score over 85. Then the circRNA-miRNA interactions were used to draw an interaction network (Figure 5 and Supplementary Table 7). We noticed several miRNAs which have been reported to regulate adipogenesis were included in the network, such as miR17, miR-30c, and miR-130. These results indicating that these circRNAs may potentially regulate adipogenesis by interacting with miRNAs. However, it should be noted that those results were not obtained experimentally and future work should validate the circRNA-miRNA interactions.

## Discussion

Prior studies have described circRNAs are abundant in white adipose tissue. Arcinas et al. (2019), for example, reported that up to 6,000 and 2,000 circRNAs were detected in human adipose tissue and mouse adipose tissue respectively. Liu et al. (2020) compared the circRNA expression profiles of obese and lean individuals. They identified that circSAMD4 was highly expressed in obese individuals. However, these studies mainly focused on mature adipose tissue. Little attention has been paid to the expression profiles and functions of circRNAs in adipocytes. In the present study, we detected the expression profile of circRNAs in proliferation, pre-mature and mature stages of adipogenesis. We identified 3,711circRNAs and nearly 35% of them are novel. We noticed a dramatic change of circRNA expression profiles between the proliferation and differentiation stages. These findings confirmed that circRNAs are abundant in adipose tissue, not a by-product of splicing. In addition, we noticed the circRNA number in our study is different from the previous study, which may result from differences in cell conditions, circRNA identification methods, and sequence depth. Thus, it is essential to investigate circRNAs expression profiles in various cell lines, tissues, and developmental stages.

Several reports indicated that circRNAs can regulate the expression of their parental genes. It has been suggested that circRNAs and their linear counterparts share the same pre-RNA (Aufiero et al., 2018), thus they may compete and negatively regulate the abundance of each other. In contrast to that, other reports suggested that circRNAs can also positively regulate gene expression. Some circRNAs accumulate at promoter regions and interact with Pol II and U1 snRNP to increase transcription of their parental genes (Li et al., 2015). Another circRNA, circFECR1, can bind to its parental gene and regulate transcription by an epigenetic mechanism. CircFECR1 recruits a demethylase and induces DNA hypomethylation in CpG islands of the promoter, thus enhanced parental gene expression (Chen N. et al., 2018). We examined the correlation between individual circRNA and parental genes. We discovered that nearly 50% of circRNAs were significantly correlated with their parental genes. When we checked them in detail, we found 37% of them were positively correlated, while the other 12% were negatively correlated. The complex correlation between circRNAs and parental genes may explain why the overall correlation is very weak and not significant. Further, when we check the correlation of the DECs and their parental genes, a much higher ratio of positive correlation was found. We also noted that the circRNAs which were derived from the same parental gene show different expression trends.

The functions of circRNAs largely remained to be investigated. One clue to predict circRNA functions is their parental genes. Functional analysis showed that the parental genes of DECs were enriched in many adipogenesis and fat metabolism-related pathways, such as GnRH signaling pathway, MAPK signaling pathway, type II diabetes mellitus, and cAMP signaling pathway. We also found several parental genes of the DECs play key roles in regulating adipogenesis. Nsd2 is the parental gene of circ_0001335. Depleting Nsd2 impairs adipogenesis by increasing H3K27me3, thus preventing the induction of C/EBPα and PPARγ (Zhuang et al., 2018). The parental gene Selenbp1 is identified as an H_2_S-producing enzyme. Selenbp1 silencing downregulates H_2_S levels and inhibits adipogenesis (Randi et al., 2021). The Fkbp5 and Fndc3b are also circRNA parental genes that are essential for adipogenesis (Tominaga et al., 2004; Zhang L. et al., 2017). Another clue to predict circRNA functions is based on miRNAs. As circRNAs were reported to act as sponges to titrate the levels of miRNAs, they can regulate miRNA target genes indirectly. CircSAMD4A is highly expressed in obese people and acts as a sponge for miR-138-5p to promote adipogenesis (Liu et al., 2020). The miR-138 effectively reduces lipid droplet accumulation by targeting adipogenesis genes (Yang et al., 2011). In bovine adipose tissue, circFUT10 directly interacts with let-7c/let-e to promote adipocyte proliferation and inhibit differentiation (Jiang et al., 2020). In the current study, we noticed that DECs could interact with a great number of miRNAs and many of the miRNAs have been reported to regulate adipogenesis. We predicted that circ_0010609 may act as a sponge for miR130a which was reported to inhibit adipogenesis differentiation *via* suppressing PPARγ expression (Lee et al., 2011). The miR-30 family represents 4.9% of the miRNA reads in adipocytes and positively regulates adipogenesis (Zaragosi et al., 2011; Irani and Hussain, 2015). According to our results, circSlc10a7 and circ_0010609 contained multiple binding sites for distinct miR-30 family members, indicating the potential roles of these circRNAs in regulating adipocyte activity.

## Conclusion

In summary, we globally detected the circRNA expression profiles during adipogenesis. We concluded that circRNAs are abundant and dynamically express in adipogenesis. Nearly 50% of the circRNAs are correlated with their parental gene expression. Adipose circRNAs may be involved in adipogenesis-related pathways and act as miRNA sponges to modulate gene expression. These identified circRNAs may serve as new candidates to regulate adipogenesis and combat obesity. However, some limitations are worth noting. Although our hypotheses were supported statistically, future experimental work is needed to understand the functions of the indicated circRNAs in adipogenesis.

## Data Availability Statement

The datasets presented in this study can be found in online repositories. The names of the repository/repositories and accession number(s) can be found in the article/Supplementary Material.

## Ethics Statement

The animal study was reviewed and approved by the Xinyang Normal University Animal Care and Use Committee.

## Author Contributions

P-PZ designed the experiments and wrote the manuscript. P-PZ, Y-JX, C-CL, X-FC, and H-XX analyzed the data. P-PZ, M-XS, Y-LW, QH, and C-YD performed the experiments. All authors have read and agreed to the published version of the manuscript.

## Conflict of Interest

The authors declare that the research was conducted in the absence of any commercial or financial relationships that could be construed as a potential conflict of interest.

## Publisher’s Note

All claims expressed in this article are solely those of the authors and do not necessarily represent those of their affiliated organizations, or those of the publisher, the editors and the reviewers. Any product that may be evaluated in this article, or claim that may be made by its manufacturer, is not guaranteed or endorsed by the publisher.

## Footnotes

1
https://primer3.ut.ee/

2
http://kobas.cbi.pku.edu.cn/kobas3

3
http://www.circbase.org/

## Supplementary Material

Supplementary Figure 1 Correlation of the expression profiles of differential circRNAs and their parental genes during adipogenesis.

Supplementary Table 1 List of circRNAs identified by RNA-seq in white adipogenesis.

Supplementary Table 2 List of primers used to detect circRNAs.

Supplementary Table 3 List of differentially expressed circRNAs across different stages of adipogenesis.

Supplementary Table 4 GO terms and KEGG pathways enriched for the host genes of differentially expressed circRNAs.

Supplementary Table 5 GO terms and KEGG pathways enriched for the host genes of group 1 and group 4 differentially expressed circRNAs.

Supplementary Table 6 Correlation of the expression profiles of circRNAs and their parental genes during adipogenesis.

Supplementary Table 7 Prediction of circRNA-miRNA interactions.
