# Supplementary figures and images for: Identification of Circular RNA Expression Profiles in White Adipocytes and Their Roles in Adipogenesis
Source: Front Physiol. 2021 Aug 19;12:728208. doi: 10.3389/fphys.2021.728208 (PMC8417237; doi:10.3389/fphys.2021.728208)

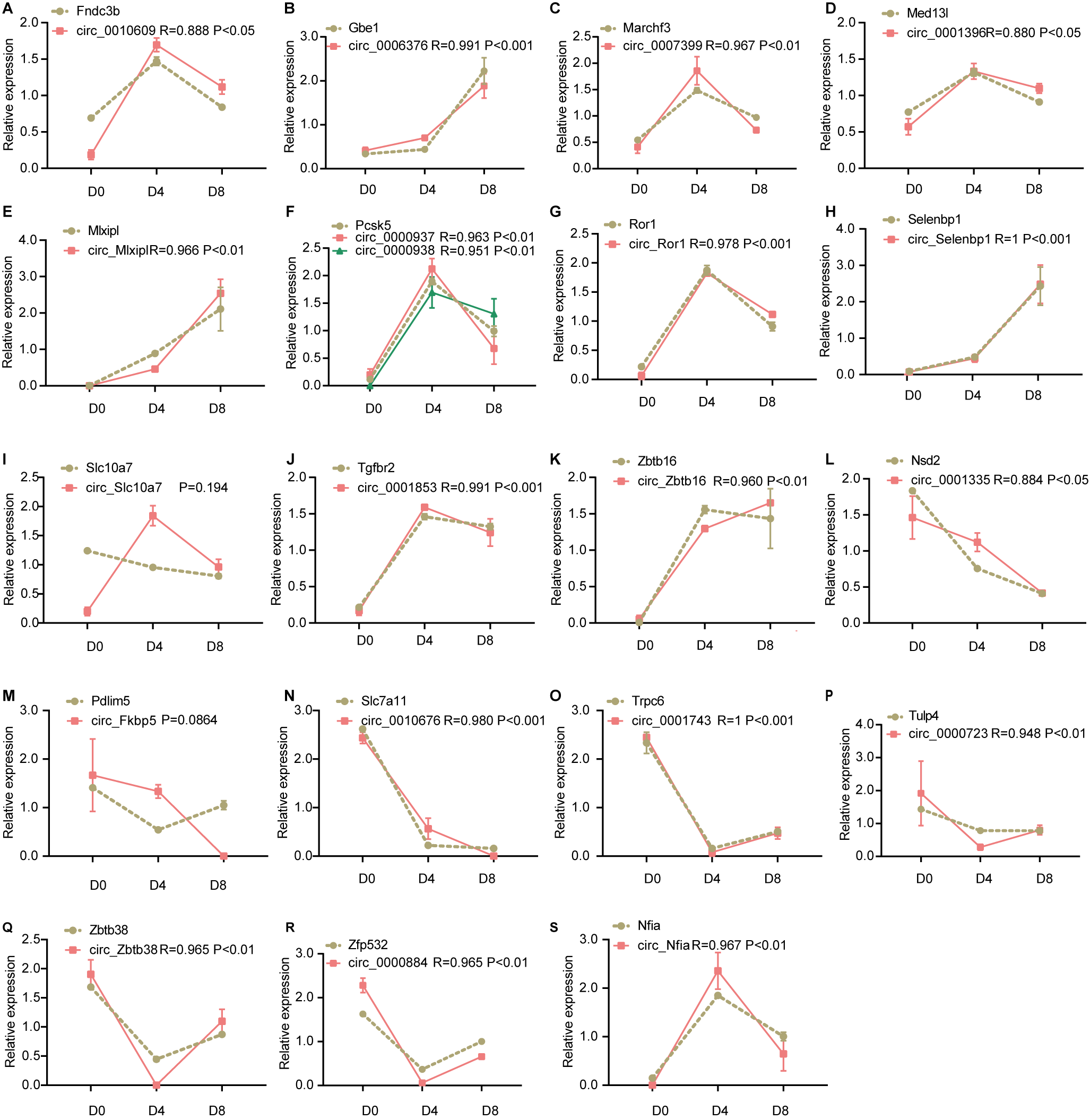

Supplement: Supplementary Figure 1 — Correlation of the expression profiles of differential circRNAs and their parental genes during adipogenesis. [file Image_1.tif]
